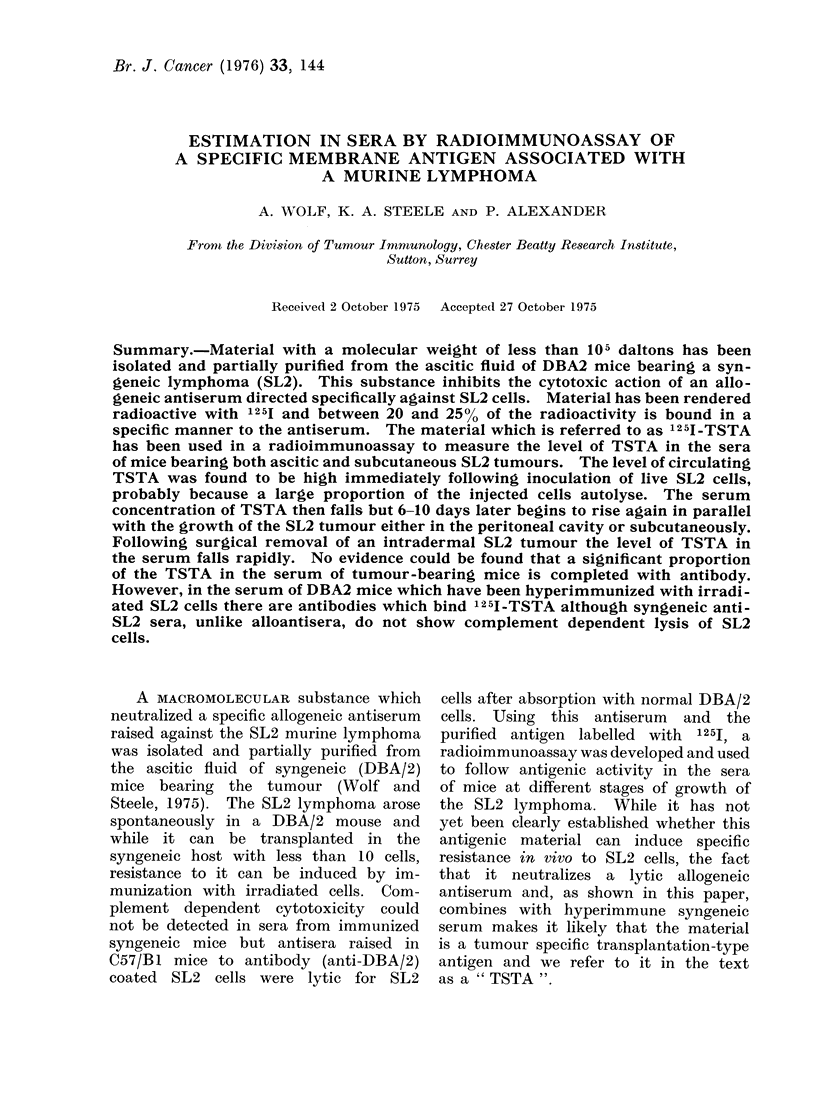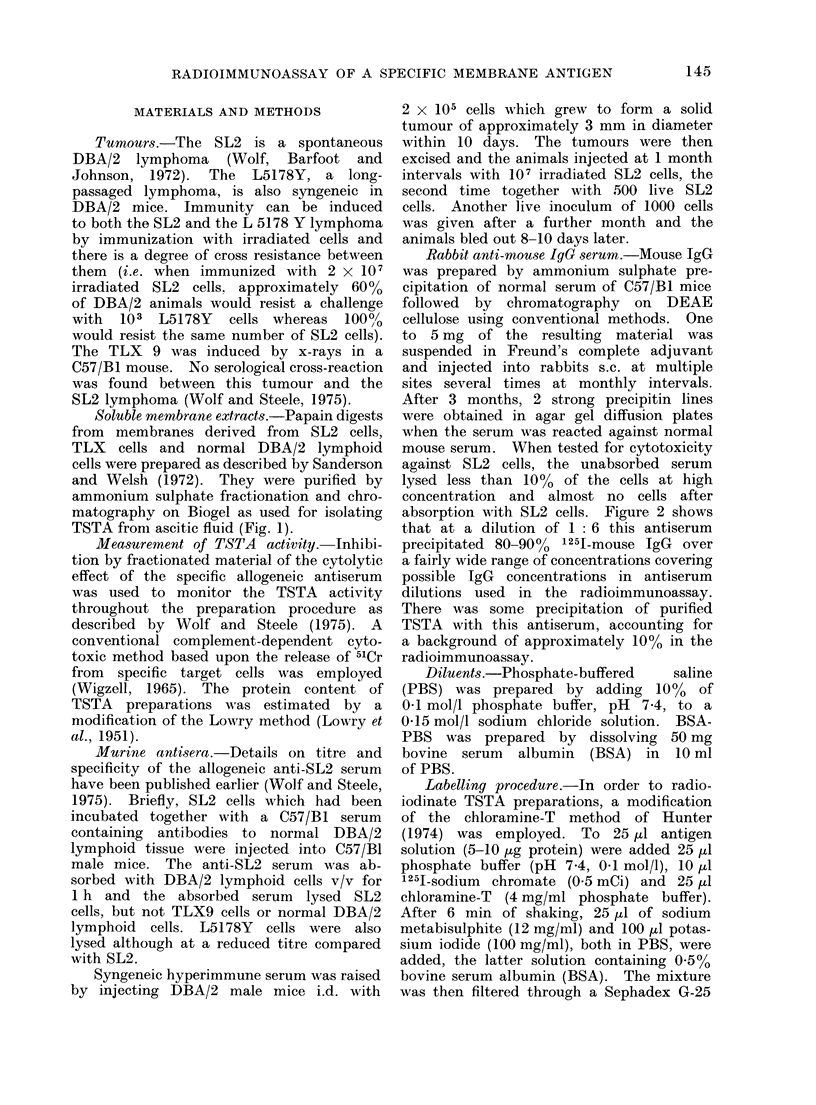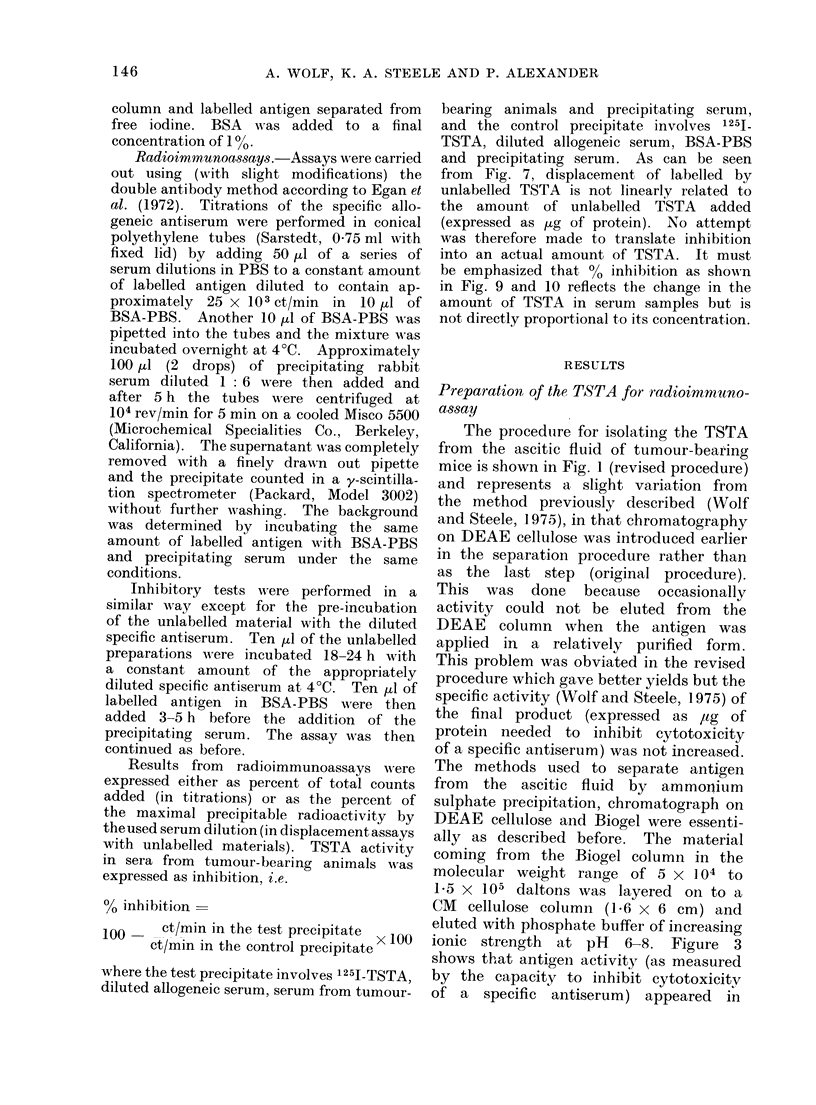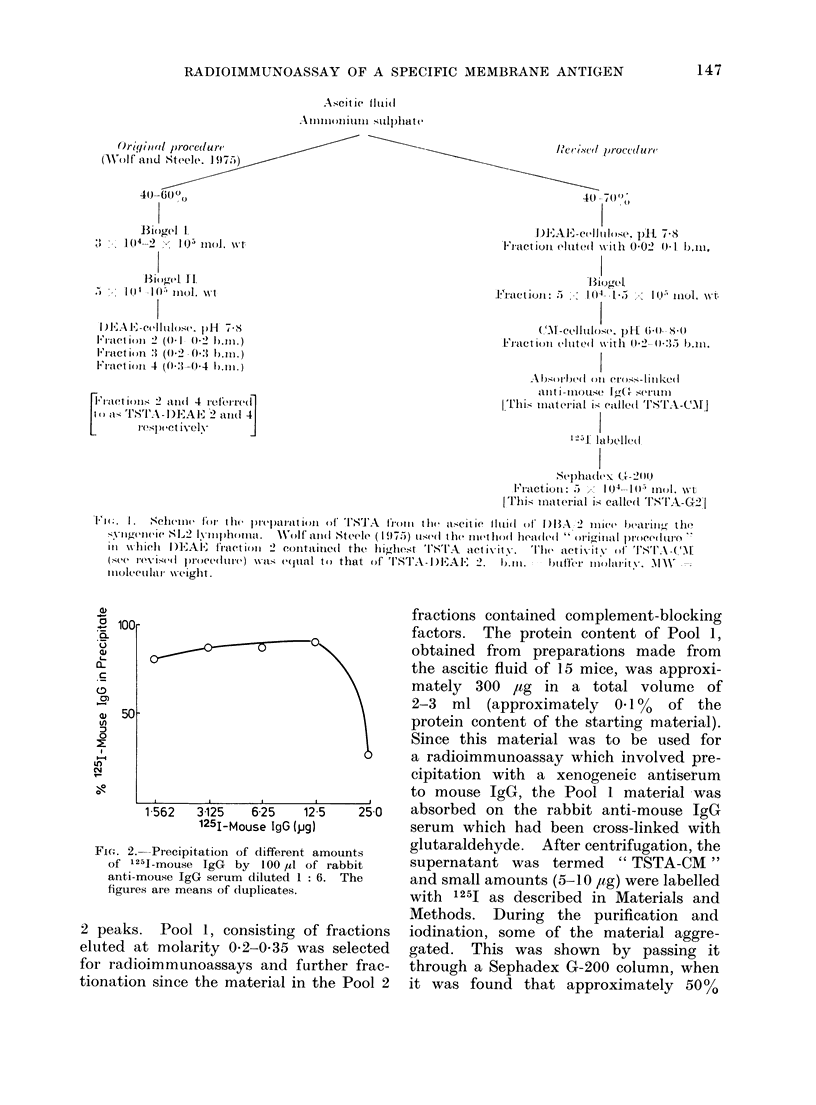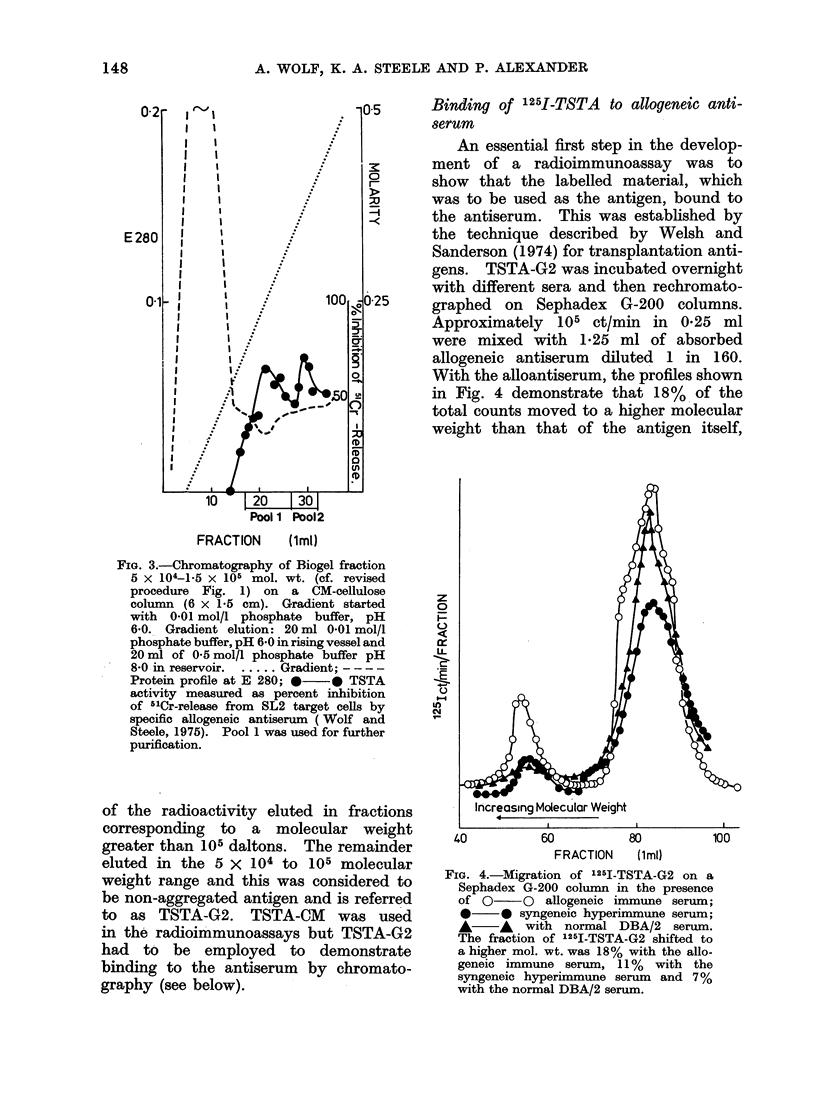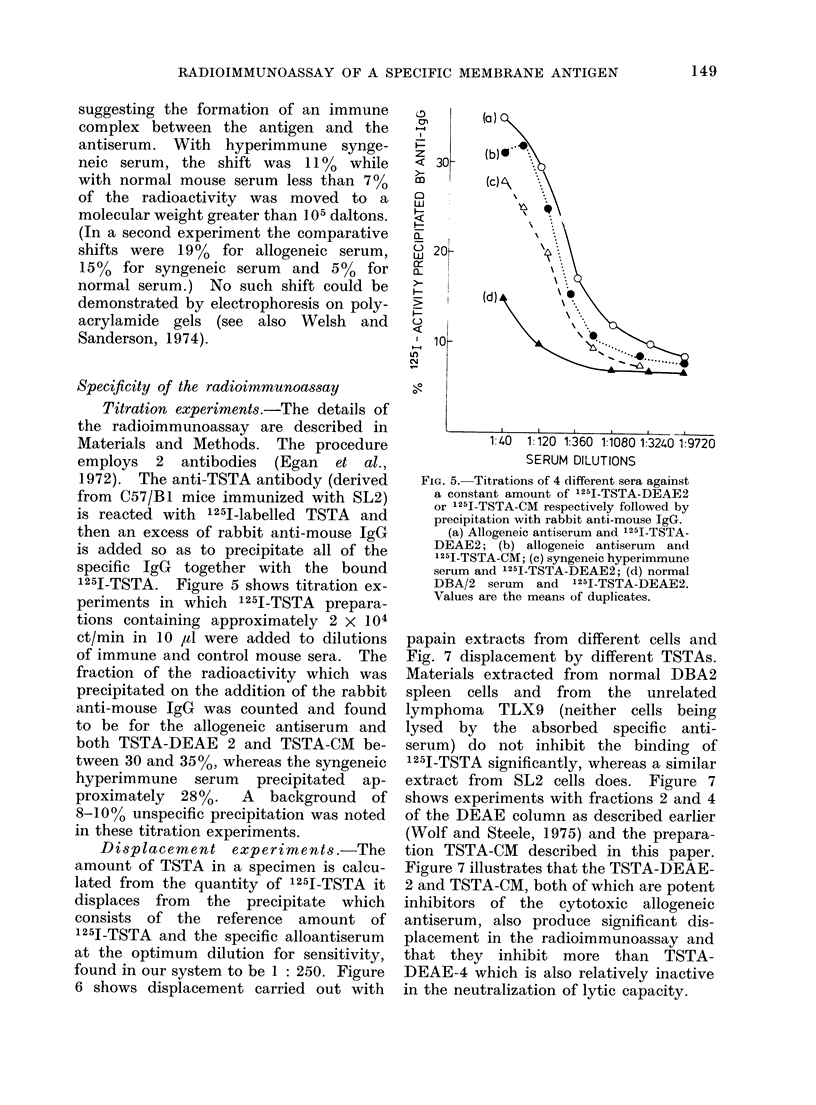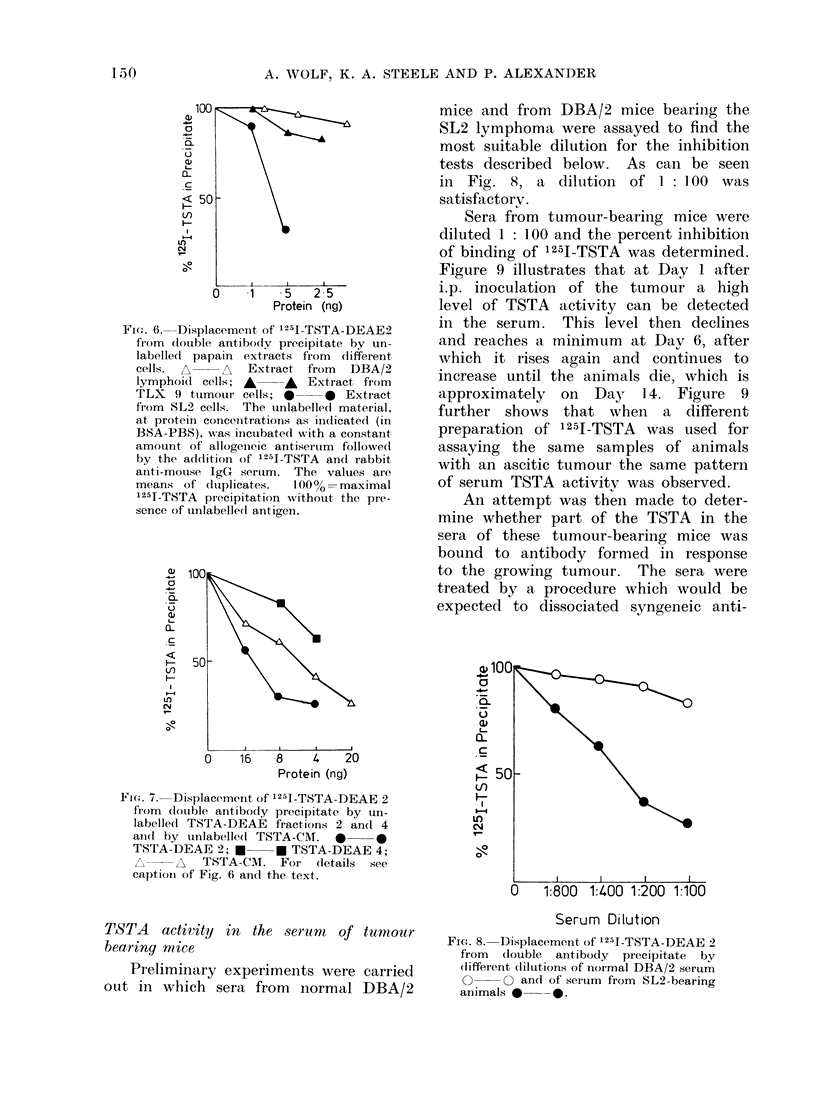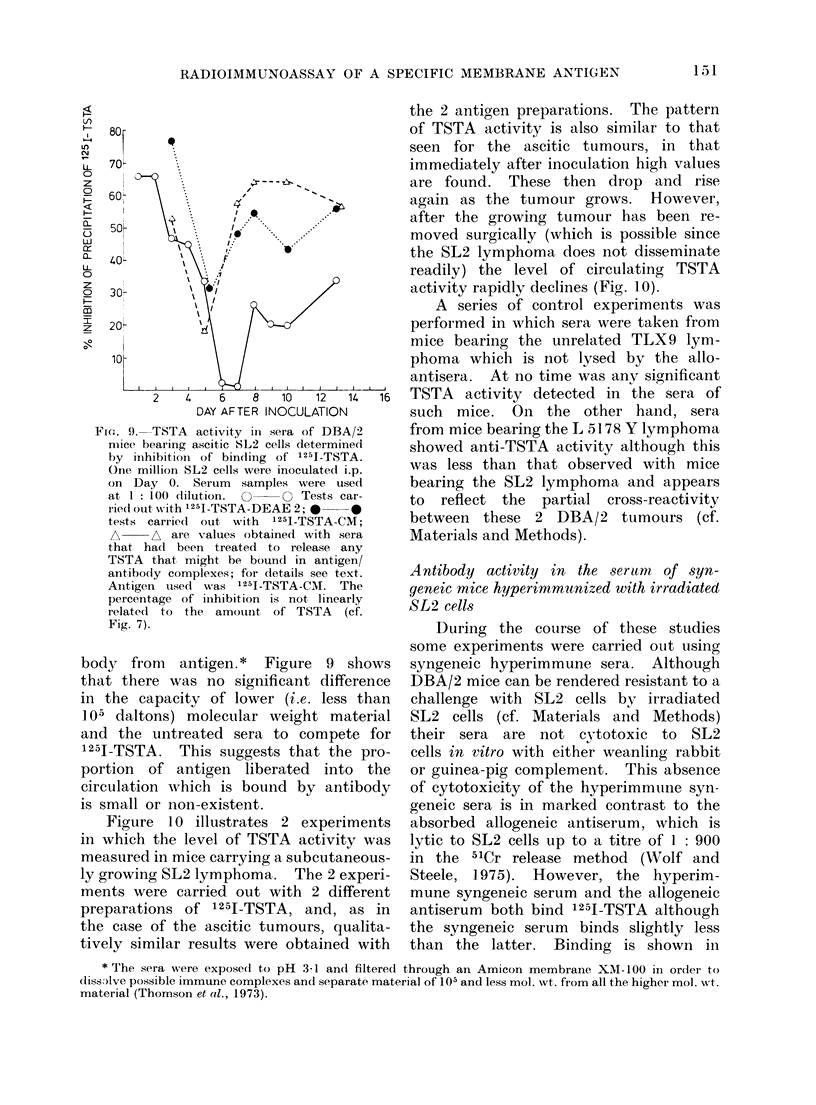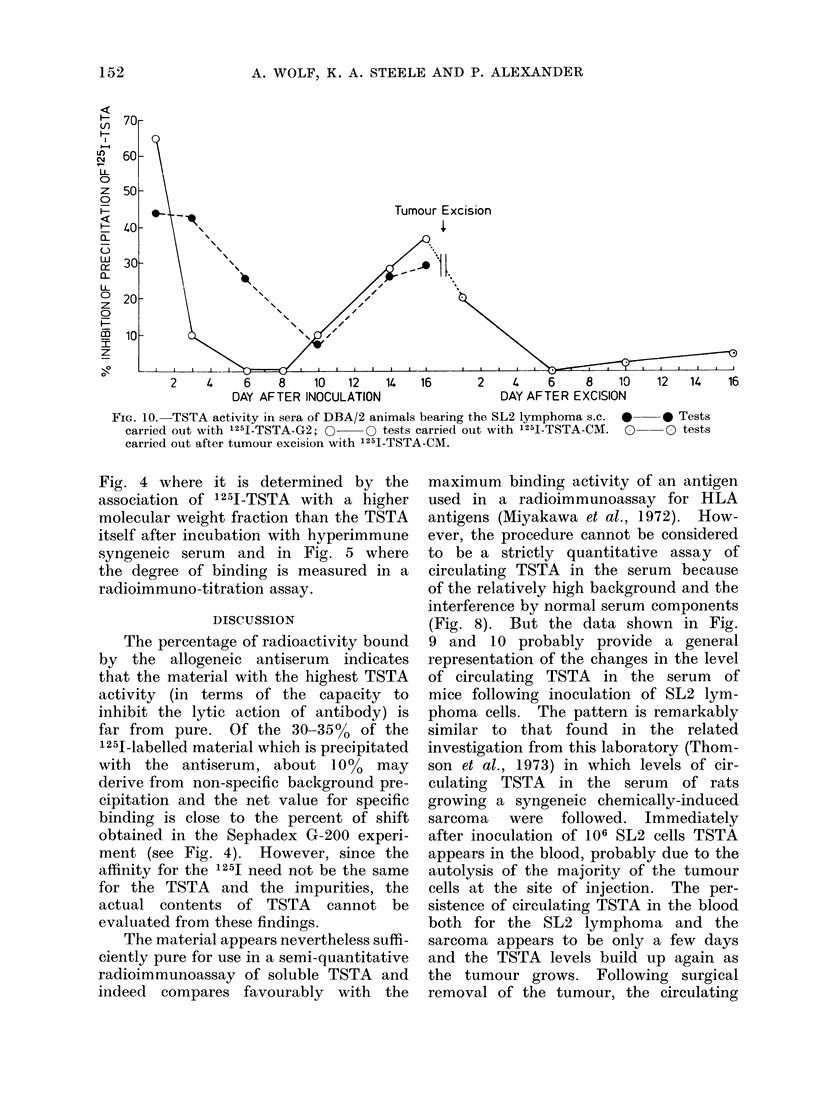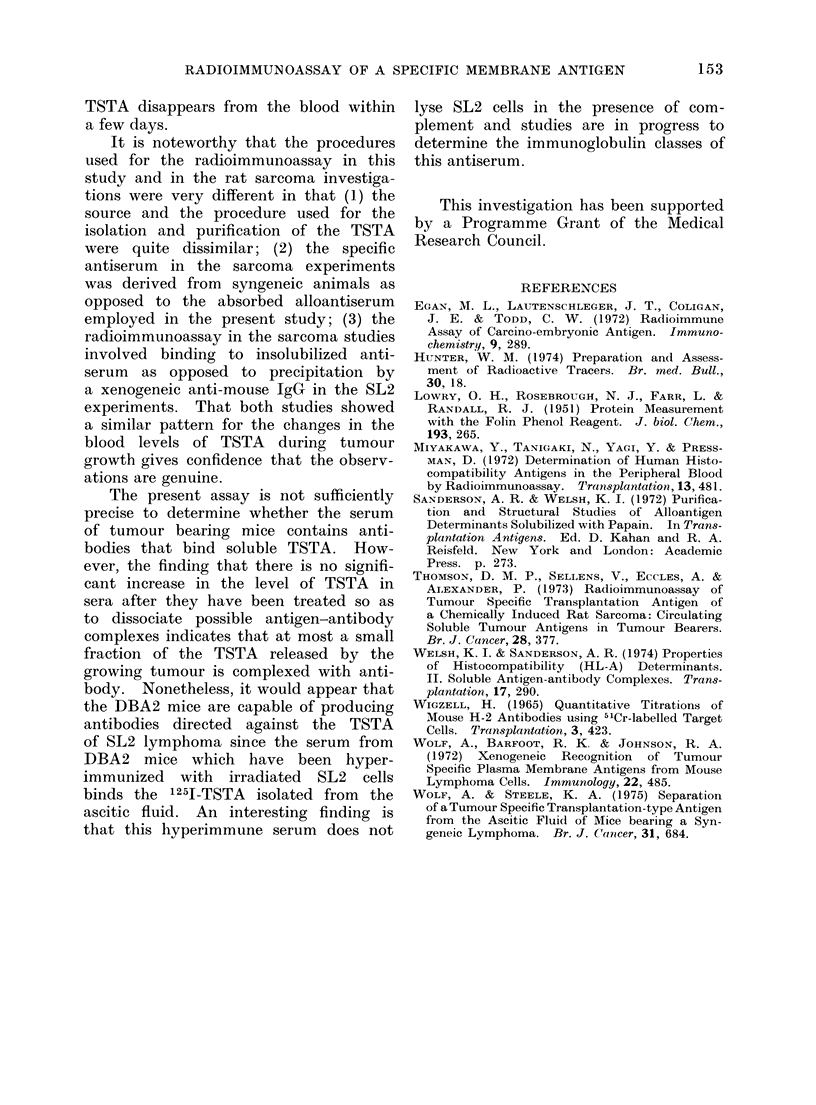# Estimation in sera by radioimmunoassay of a specific membrane antigen associated with a murine lymphoma.

**DOI:** 10.1038/bjc.1976.19

**Published:** 1976-02

**Authors:** A. Wolf, K. A. Steele, P. Alexander

## Abstract

Material with a molecular weight of less than 10(5) daltons has been isolated and partially purified from the ascitic fluid of DBA2 mice bearing a syngeneic lymphoma (SL2). This substance inhibits the cytotoxic action of an allogeneic antiserum directed specifically against SL2 cells. Material has been rendered radioactive with 125I and between 20 and 25% of the radioactivity is bound in a specific manner to the antiserum. The material which is referred to as 125I-TSTA has been used in a radioimmunoassay to measure the level of TSTA in the sera of mice bearing both ascitic and subcutaneous SL2 tumours. The level of circulating TSTA was found to be high immediatley following inoculation of live SL2 cells, probably because a large proportion of the injected cells autolyse. The serum concentration of TSTA then falls but 6-10 days later begins to rise again in parallel with the growth of the SL2 tumour either in the peritoneal cavity or subcutaneously. Following surgical removal of an intradermal SL2 tumour the level of TSTA in the serum falls rapidly. No evidence could be found that a significant proportion of the TSTA in the serum of tumour-bearing mice is completed with antibody. However, in the serum of DBA2 mice which have been hyperimmunized with irradiated SL2 cells there are antibodies which bind 125I-TSTA although syngeneic anti-SL2 sera, unlike alloantisera, do not show complement dependent lysis of SL2 cells.


*Br. J. Cancer* (1976) **33**, 144

### ESTIMATION IN SERA BY RADIOIMMUNOASSAY OF A SPECIFIC MEMBRANE ANTIGEN ASSOCIATED WITH A MURINE LYMPHOMA

A. WOLF, K. A. STEELE AND P. ALEXANDER

*From the Division of Tumour Immunology, Chester Beatty Research Institute, Sutton, Surrey*

Received 2 October 1975 Accepted 27 October 1975

**Summary.—Material with a molecular weight of less than $10^5$ daltons has been isolated and partially purified from the ascitic fluid of DBA2 mice bearing a syngeneic lymphoma (SL2). This substance inhibits the cytotoxic action of an allogeneic antiserum directed specifically against SL2 cells. Material has been rendered radioactive with $^{125}$I and between 20 and 25% of the radioactivity is bound in a specific manner to the antiserum. The material which is referred to as $^{125}$I-TSTA has been used in a radioimmunoassay to measure the level of TSTA in the sera of mice bearing both ascitic and subcutaneous SL2 tumours. The level of circulating TSTA was found to be high immediately following inoculation of live SL2 cells, probably because a large proportion of the injected cells autolyse. The serum concentration of TSTA then falls but 6–10 days later begins to rise again in parallel with the growth of the SL2 tumour either in the peritoneal cavity or subcutaneously. Following surgical removal of an intradermal SL2 tumour the level of TSTA in the serum falls rapidly. No evidence could be found that a significant proportion of the TSTA in the serum of tumour-bearing mice is completed with antibody. However, in the serum of DBA2 mice which have been hyperimmunized with irradiated SL2 cells there are antibodies which bind $^{125}$I-TSTA although syngeneic anti-SL2 sera, unlike alloantisera, do not show complement dependent lysis of SL2 cells.**

A MACROMOLECULAR substance which neutralized a specific allogeneic antiserum raised against the SL2 murine lymphoma was isolated and partially purified from the ascitic fluid of syngeneic (DBA/2) mice bearing the tumour (Wolf and Steele, 1975). The SL2 lymphoma arose spontaneously in a DBA/2 mouse and while it can be transplanted in the syngeneic host with less than 10 cells, resistance to it can be induced by immunization with irradiated cells. Complement dependent cytotoxicity could not be detected in sera from immunized syngeneic mice but antisera raised in C57/Bl mice to antibody (anti-DBA/2) coated SL2 cells were lytic for SL2 cells after absorption with normal DBA/2 cells. Using this antiserum and the purified antigen labelled with $^{125}$I, a radioimmunoassay was developed and used to follow antigenic activity in the sera of mice at different stages of growth of the SL2 lymphoma. While it has not yet been clearly established whether this antigenic material can induce specific resistance *in vivo* to SL2 cells, the fact that it neutralizes a lytic allogeneic antiserum and, as shown in this paper, combines with hyperimmune syngeneic serum makes it likely that the material is a tumour specific transplantation-type antigen and we refer to it in the text as a " TSTA ".

### MATERIALS AND METHODS

*Tumours.*—The SL2 is a spontaneous DBA/2 lymphoma (Wolf, Barfoot and Johnson, 1972). The L5178Y, a long-passaged lymphoma, is also syngeneic in DBA/2 mice. Immunity can be induced to both the SL2 and the L 5178 Y lymphoma by immunization with irradiated cells and there is a degree of cross resistance between them (*i.e.* when immunized with $2 \times 10^7$ irradiated SL2 cells, approximately 60% of DBA/2 animals would resist a challenge with $10^3$ L5178Y cells whereas 100% would resist the same number of SL2 cells). The TLX 9 was induced by x-rays in a C57/Bl mouse. No serological cross-reaction was found between this tumour and the SL2 lymphoma (Wolf and Steele, 1975).

*Soluble membrane extracts.*—Papain digests from membranes derived from SL2 cells, TLX cells and normal DBA/2 lymphoid cells were prepared as described by Sanderson and Welsh (1972). They were purified by ammonium sulphate fractionation and chromatography on Biogel as used for isolating TSTA from ascitic fluid (Fig. 1).

*Measurement of TSTA activity.*—Inhibition by fractionated material of the cytolytic effect of the specific allogeneic antiserum was used to monitor the TSTA activity throughout the preparation procedure as described by Wolf and Steele (1975). A conventional complement-dependent cytotoxic method based upon the release of $^{51}$Cr from specific target cells was employed (Wigzell, 1965). The protein content of TSTA preparations was estimated by a modification of the Lowry method (Lowry *et al.*, 1951).

*Murine antisera.*—Details on titre and specificity of the allogeneic anti-SL2 serum have been published earlier (Wolf and Steele, 1975). Briefly, SL2 cells which had been incubated together with a C57/Bl serum containing antibodies to normal DBA/2 lymphoid tissue were injected into C57/Bl male mice. The anti-SL2 serum was absorbed with DBA/2 lymphoid cells v/v for 1 h and the absorbed serum lysed SL2 cells, but not TLX9 cells or normal DBA/2 lymphoid cells. L5178Y cells were also lysed although at a reduced titre compared with SL2.

Syngeneic hyperimmune serum was raised by injecting DBA/2 male mice i.d. with $2 \times 10^5$ cells which grew to form a solid tumour of approximately 3 mm in diameter within 10 days. The tumours were then excised and the animals injected at 1 month intervals with $10^7$ irradiated SL2 cells, the second time together with 500 live SL2 cells. Another live inoculum of 1000 cells was given after a further month and the animals bled out 8–10 days later.

*Rabbit anti-mouse IgG serum.*—Mouse IgG was prepared by ammonium sulphate precipitation of normal serum of C57/Bl mice followed by chromatography on DEAE cellulose using conventional methods. One to 5 mg of the resulting material was suspended in Freund's complete adjuvant and injected into rabbits s.c. at multiple sites several times at monthly intervals. After 3 months, 2 strong precipitin lines were obtained in agar gel diffusion plates when the serum was reacted against normal mouse serum. When tested for cytotoxicity against SL2 cells, the unabsorbed serum lysed less than 10% of the cells at high concentration and almost no cells after absorption with SL2 cells. Figure 2 shows that at a dilution of 1 : 6 this antiserum precipitated 80–90% $^{125}$I-mouse IgG over a fairly wide range of concentrations covering possible IgG concentrations in antiserum dilutions used in the radioimmunoassay. There was some precipitation of purified TSTA with this antiserum, accounting for a background of approximately 10% in the radioimmunoassay.

*Diluents.*—Phosphate-buffered saline (PBS) was prepared by adding 10% of 0·1 mol/l phosphate buffer, pH 7·4, to a 0·15 mol/l sodium chloride solution. BSA-PBS was prepared by dissolving 50 mg bovine serum albumin (BSA) in 10 ml of PBS.

*Labelling procedure.*—In order to radioiodinate TSTA preparations, a modification of the chloramine-T method of Hunter (1974) was employed. To 25 $\mu$l antigen solution (5–10 $\mu$g protein) were added 25 $\mu$l phosphate buffer (pH 7·4, 0·1 mol/l), 10 $\mu$l $^{125}$I-sodium chromate (0·5 mCi) and 25 $\mu$l chloramine-T (4 mg/ml phosphate buffer). After 6 min of shaking, 25 $\mu$l of sodium metabisulphite (12 mg/ml) and 100 $\mu$l potassium iodide (100 mg/ml), both in PBS, were added, the latter solution containing 0·5% bovine serum albumin (BSA). The mixture was then filtered through a Sephadex G-25

column and labelled antigen separated from free iodine. BSA was added to a final concentration of 1%.

*Radioimmunoassays.*—Assays were carried out using (with slight modifications) the double antibody method according to Egan *et al.* (1972). Titrations of the specific allogeneic antiserum were performed in conical polyethylene tubes (Sarstedt, 0·75 ml with fixed lid) by adding 50 $\mu$l of a series of serum dilutions in PBS to a constant amount of labelled antigen diluted to contain approximately $25 \times 10^3$ ct/min in 10 $\mu$l of BSA-PBS. Another 10 $\mu$l of BSA-PBS was pipetted into the tubes and the mixture was incubated overnight at 4 °C. Approximately 100 $\mu$l (2 drops) of precipitating rabbit serum diluted 1 : 6 were then added and after 5 h the tubes were centrifuged at $10^4$ rev/min for 5 min on a cooled Misco 5500 (Microchemical Specialities Co., Berkeley, California). The supernatant was completely removed with a finely drawn out pipette and the precipitate counted in a $\gamma$-scintillation spectrometer (Packard, Model 3002) without further washing. The background was determined by incubating the same amount of labelled antigen with BSA-PBS and precipitating serum under the same conditions.

Inhibitory tests were performed in a similar way except for the pre-incubation of the unlabelled material with the diluted specific antiserum. Ten $\mu$l of the unlabelled preparations were incubated 18–24 h with a constant amount of the appropriately diluted specific antiserum at 4 °C. Ten $\mu$l of labelled antigen in BSA-PBS were then added 3–5 h before the addition of the precipitating serum. The assay was then continued as before.

Results from radioimmunoassays were expressed either as percent of total counts added (in titrations) or as the percent of the maximal precipitable radioactivity by the used serum dilution (in displacement assays with unlabelled materials). TSTA activity in sera from tumour-bearing animals was expressed as inhibition, *i.e.*

$$\% \text{ inhibition} = 100 - \frac{\text{ct/min in the test precipitate}}{\text{ct/min in the control precipitate}} \times 100$$

where the test precipitate involves $^{125}$I-TSTA, diluted allogeneic serum, serum from tumour-bearing animals and precipitating serum, and the control precipitate involves $^{125}$I-TSTA, diluted allogeneic serum, BSA-PBS and precipitating serum. As can be seen from Fig. 7, displacement of labelled by unlabelled TSTA is not linearly related to the amount of unlabelled TSTA added (expressed as $\mu$g of protein). No attempt was therefore made to translate inhibition into an actual amount of TSTA. It must be emphasized that % inhibition as shown in Fig. 9 and 10 reflects the change in the amount of TSTA in serum samples but is not directly proportional to its concentration.

### RESULTS

### *Preparation of the TSTA for radioimmunoassay*

The procedure for isolating the TSTA from the ascitic fluid of tumour-bearing mice is shown in Fig. 1 (revised procedure) and represents a slight variation from the method previously described (Wolf and Steele, 1975), in that chromatography on DEAE cellulose was introduced earlier in the separation procedure rather than as the last step (original procedure). This was done because occasionally activity could not be eluted from the DEAE column when the antigen was applied in a relatively purified form. This problem was obviated in the revised procedure which gave better yields but the specific activity (Wolf and Steele, 1975) of the final product (expressed as $\mu$g of protein needed to inhibit cytotoxicity of a specific antiserum) was not increased. The methods used to separate antigen from the ascitic fluid by ammonium sulphate precipitation, chromatograph on DEAE cellulose and Biogel were essentially as described before. The material coming from the Biogel column in the molecular weight range of $5 \times 10^4$ to $1{\cdot}5 \times 10^5$ daltons was layered on to a CM cellulose column (1·6 × 6 cm) and eluted with phosphate buffer of increasing ionic strength at pH 6–8. Figure 3 shows that antigen activity (as measured by the capacity to inhibit cytotoxicity of a specific antiserum) appeared in

FIG. 1. Scheme for the preparation of TSTA from the ascitic fluid of DBA/2 mice bearing the syngeneic SL2 lymphoma. Wolf and Steele (1975) used the method headed "original procedure" in which DEAE fraction 2 contained the highest TSTA activity. The activity of TSTA-CM (see revised procedure) was equal to that of TSTA-DEAE 2. b.m. = buffer molarity, MW = molecular weight.

FIG. 2.—Precipitation of different amounts of $^{125}$I-mouse IgG by 100 $\mu$l of rabbit anti-mouse IgG serum diluted 1 : 6. The figures are means of duplicates.

2 peaks. Pool 1, consisting of fractions eluted at molarity 0·2–0·35 was selected for radioimmunoassays and further fractionation since the material in the Pool 2 fractions contained complement-blocking factors. The protein content of Pool 1, obtained from preparations made from the ascitic fluid of 15 mice, was approximately 300 $\mu$g in a total volume of 2–3 ml (approximately 0·1% of the protein content of the starting material). Since this material was to be used for a radioimmunoassay which involved precipitation with a xenogeneic antiserum to mouse IgG, the Pool 1 material was absorbed on the rabbit anti-mouse IgG serum which had been cross-linked with glutaraldehyde. After centrifugation, the supernatant was termed "TSTA-CM" and small amounts (5–10 $\mu$g) were labelled with $^{125}$I as described in Materials and Methods. During the purification and iodination, some of the material aggregated. This was shown by passing it through a Sephadex G-200 column, when it was found that approximately 50%

FIG. 3.—Chromatography of Biogel fraction $5 \times 10^4$–$1 \cdot 5 \times 10^5$ mol. wt. (cf. revised procedure Fig. 1) on a CM-cellulose column ($6 \times 1 \cdot 5$ cm). Gradient started with 0·01 mol/l phosphate buffer, pH 6·0. Gradient elution: 20 ml 0·01 mol/l phosphate buffer, pH 6·0 in rising vessel and 20 ml of 0·5 mol/l phosphate buffer pH 8·0 in reservoir. ..... Gradient; – – – – Protein profile at E 280; ●——● TSTA activity measured as percent inhibition of $^{51}$Cr-release from SL2 target cells by specific allogeneic antiserum (Wolf and Steele, 1975). Pool 1 was used for further purification.

of the radioactivity eluted in fractions corresponding to a molecular weight greater than $10^5$ daltons. The remainder eluted in the $5 \times 10^4$ to $10^5$ molecular weight range and this was considered to be non-aggregated antigen and is referred to as TSTA-G2. TSTA-CM was used in the radioimmunoassays but TSTA-G2 had to be employed to demonstrate binding to the antiserum by chromatography (see below).

*Binding of $^{125}$I-TSTA to allogeneic antiserum*

An essential first step in the development of a radioimmunoassay was to show that the labelled material, which was to be used as the antigen, bound to the antiserum. This was established by the technique described by Welsh and Sanderson (1974) for transplantation antigens. TSTA-G2 was incubated overnight with different sera and then rechromatographed on Sephadex G-200 columns. Approximately $10^5$ ct/min in 0·25 ml were mixed with 1·25 ml of absorbed allogeneic antiserum diluted 1 in 160. With the alloantiserum, the profiles shown in Fig. 4 demonstrate that 18% of the total counts moved to a higher molecular weight than that of the antigen itself,

FIG. 4.—Migration of $^{125}$I-TSTA-G2 on a Sephadex G-200 column in the presence of ○——○ allogeneic immune serum; ●——● syngeneic hyperimmune serum; ▲——▲ with normal DBA/2 serum. The fraction of $^{125}$I-TSTA-G2 shifted to a higher mol. wt. was 18% with the allogeneic immune serum, 11% with the syngeneic hyperimmune serum and 7% with the normal DBA/2 serum.

suggesting the formation of an immune complex between the antigen and the antiserum. With hyperimmune syngeneic serum, the shift was 11% while with normal mouse serum less than 7% of the radioactivity was moved to a molecular weight greater than $10^5$ daltons. (In a second experiment the comparative shifts were 19% for allogeneic serum, 15% for syngeneic serum and 5% for normal serum.) No such shift could be demonstrated by electrophoresis on polyacrylamide gels (see also Welsh and Sanderson, 1974).

*Specificity of the radioimmunoassay*

*Titration experiments*.—The details of the radioimmunoassay are described in Materials and Methods. The procedure employs 2 antibodies (Egan *et al.*, 1972). The anti-TSTA antibody (derived from C57/Bl mice immunized with SL2) is reacted with $^{125}$I-labelled TSTA and then an excess of rabbit anti-mouse IgG is added so as to precipitate all of the specific IgG together with the bound $^{125}$I-TSTA. Figure 5 shows titration experiments in which $^{125}$I-TSTA preparations containing approximately $2 \times 10^4$ ct/min in 10 $\mu$l were added to dilutions of immune and control mouse sera. The fraction of the radioactivity which was precipitated on the addition of the rabbit anti-mouse IgG was counted and found to be for the allogeneic antiserum and both TSTA-DEAE 2 and TSTA-CM between 30 and 35%, whereas the syngeneic hyperimmune serum precipitated approximately 28%. A background of 8–10% unspecific precipitation was noted in these titration experiments.

FIG. 5.—Titrations of 4 different sera against a constant amount of $^{125}$I-TSTA-DEAE2 or $^{125}$I-TSTA-CM respectively followed by precipitation with rabbit anti-mouse IgG. (a) Allogeneic antiserum and $^{125}$I-TSTA-DEAE2; (b) allogeneic antiserum and $^{125}$I-TSTA-CM; (c) syngeneic hyperimmune serum and $^{125}$I-TSTA-DEAE2; (d) normal DBA/2 serum and $^{125}$I-TSTA-DEAE2. Values are the means of duplicates.

*Displacement experiments*.—The amount of TSTA in a specimen is calculated from the quantity of $^{125}$I-TSTA it displaces from the precipitate which consists of the reference amount of $^{125}$I-TSTA and the specific alloantiserum at the optimum dilution for sensitivity, found in our system to be 1 : 250. Figure 6 shows displacement carried out with papain extracts from different cells and Fig. 7 displacement by different TSTAs. Materials extracted from normal DBA2 spleen cells and from the unrelated lymphoma TLX9 (neither cells being lysed by the absorbed specific antiserum) do not inhibit the binding of $^{125}$I-TSTA significantly, whereas a similar extract from SL2 cells does. Figure 7 shows experiments with fractions 2 and 4 of the DEAE column as described earlier (Wolf and Steele, 1975) and the preparation TSTA-CM described in this paper. Figure 7 illustrates that the TSTA-DEAE-2 and TSTA-CM, both of which are potent inhibitors of the cytotoxic allogeneic antiserum, also produce significant displacement in the radioimmunoassay and that they inhibit more than TSTA-DEAE-4 which is also relatively inactive in the neutralization of lytic capacity.

FIG. 6.—Displacement of $^{125}$I-TSTA-DEAE2 from double antibody precipitate by unlabelled papain extracts from different cells. △——△ Extract from DBA/2 lymphoid cells; ▲——▲ Extract from TLX 9 tumour cells; ●——● Extract from SL2 cells. The unlabelled material, at protein concentrations as indicated (in BSA-PBS), was incubated with a constant amount of allogeneic antiserum followed by the addition of $^{125}$I-TSTA and rabbit anti-mouse IgG serum. The values are means of duplicates. 100% = maximal $^{125}$I-TSTA precipitation without the presence of unlabelled antigen.

FIG. 7.—Displacement of $^{125}$I-TSTA-DEAE 2 from double antibody precipitate by unlabelled TSTA-DEAE fractions 2 and 4 and by unlabelled TSTA-CM. ●——● TSTA-DEAE 2; ■——■ TSTA-DEAE 4; △——△ TSTA-CM. For details see caption of Fig. 6 and the text.

### *TSTA activity in the serum of tumour bearing mice*

Preliminary experiments were carried out in which sera from normal DBA/2 mice and from DBA/2 mice bearing the SL2 lymphoma were assayed to find the most suitable dilution for the inhibition tests described below. As can be seen in Fig. 8, a dilution of 1 : 100 was satisfactory.

Sera from tumour-bearing mice were diluted 1 : 100 and the percent inhibition of binding of $^{125}$I-TSTA was determined. Figure 9 illustrates that at Day 1 after i.p. inoculation of the tumour a high level of TSTA activity can be detected in the serum. This level then declines and reaches a minimum at Day 6, after which it rises again and continues to increase until the animals die, which is approximately on Day 14. Figure 9 further shows that when a different preparation of $^{125}$I-TSTA was used for assaying the same samples of animals with an ascitic tumour the same pattern of serum TSTA activity was observed.

An attempt was then made to determine whether part of the TSTA in the sera of these tumour-bearing mice was bound to antibody formed in response to the growing tumour. The sera were treated by a procedure which would be expected to dissociated syngeneic anti-

FIG. 8.—Displacement of $^{125}$I-TSTA-DEAE 2 from double antibody precipitate by different dilutions of normal DBA/2 serum ○——○ and of serum from SL2-bearing animals ●——●.

FIG. 9.—TSTA activity in sera of DBA/2 mice bearing ascitic SL2 cells determined by inhibition of binding of $^{125}$I-TSTA. One million SL2 cells were inoculated i.p. on Day 0. Serum samples were used at 1 : 100 dilution. ○——○ Tests carried out with $^{125}$I-TSTA-DEAE 2; ●——● tests carried out with $^{125}$I-TSTA-CM; △——△ are values obtained with sera that had been treated to release any TSTA that might be bound in antigen/antibody complexes; for details see text. Antigen used was $^{125}$I-TSTA-CM. The percentage of inhibition is not linearly related to the amount of TSTA (cf. Fig. 7).

body from antigen.* Figure 9 shows that there was no significant difference in the capacity of lower (*i.e.* less than $10^5$ daltons) molecular weight material and the untreated sera to compete for $^{125}$I-TSTA. This suggests that the proportion of antigen liberated into the circulation which is bound by antibody is small or non-existent.

Figure 10 illustrates 2 experiments in which the level of TSTA activity was measured in mice carrying a subcutaneously growing SL2 lymphoma. The 2 experiments were carried out with 2 different preparations of $^{125}$I-TSTA, and, as in the case of the ascitic tumours, qualitatively similar results were obtained with the 2 antigen preparations. The pattern of TSTA activity is also similar to that seen for the ascitic tumours, in that immediately after inoculation high values are found. These then drop and rise again as the tumour grows. However, after the growing tumour has been removed surgically (which is possible since the SL2 lymphoma does not disseminate readily) the level of circulating TSTA activity rapidly declines (Fig. 10).

A series of control experiments was performed in which sera were taken from mice bearing the unrelated TLX9 lymphoma which is not lysed by the allo-antisera. At no time was any significant TSTA activity detected in the sera of such mice. On the other hand, sera from mice bearing the L 5178 Y lymphoma showed anti-TSTA activity although this was less than that observed with mice bearing the SL2 lymphoma and appears to reflect the partial cross-reactivity between these 2 DBA/2 tumours (cf. Materials and Methods).

*Antibody activity in the serum of syngeneic mice hyperimmunized with irradiated SL2 cells*

During the course of these studies some experiments were carried out using syngeneic hyperimmune sera. Although DBA/2 mice can be rendered resistant to a challenge with SL2 cells by irradiated SL2 cells (cf. Materials and Methods) their sera are not cytotoxic to SL2 cells *in vitro* with either weanling rabbit or guinea-pig complement. This absence of cytotoxicity of the hyperimmune syngeneic sera is in marked contrast to the absorbed allogeneic antiserum, which is lytic to SL2 cells up to a titre of 1 : 900 in the $^{51}$Cr release method (Wolf and Steele, 1975). However, the hyperimmune syngeneic serum and the allogeneic antiserum both bind $^{125}$I-TSTA although the syngeneic serum binds slightly less than the latter. Binding is shown in

* The sera were exposed to pH 3·1 and filtered through an Amicon membrane XM-100 in order to dissolve possible immune complexes and separate material of $10^5$ and less mol. wt. from all the higher mol. wt. material (Thomson *et al.*, 1973).

FIG. 10.—TSTA activity in sera of DBA/2 animals bearing the SL2 lymphoma s.c. ●——● Tests carried out with $^{125}$I-TSTA-G2; ○——○ tests carried out with $^{125}$I-TSTA-CM. ○——○ tests carried out after tumour excision with $^{125}$I-TSTA-CM.

Fig. 4 where it is determined by the association of $^{125}$I-TSTA with a higher molecular weight fraction than the TSTA itself after incubation with hyperimmune syngeneic serum and in Fig. 5 where the degree of binding is measured in a radioimmuno-titration assay.

### DISCUSSION

The percentage of radioactivity bound by the allogeneic antiserum indicates that the material with the highest TSTA activity (in terms of the capacity to inhibit the lytic action of antibody) is far from pure. Of the 30–35% of the $^{125}$I-labelled material which is precipitated with the antiserum, about 10% may derive from non-specific background precipitation and the net value for specific binding is close to the percent of shift obtained in the Sephadex G-200 experiment (see Fig. 4). However, since the affinity for the $^{125}$I need not be the same for the TSTA and the impurities, the actual contents of TSTA cannot be evaluated from these findings.

The material appears nevertheless sufficiently pure for use in a semi-quantitative radioimmunoassay of soluble TSTA and indeed compares favourably with the maximum binding activity of an antigen used in a radioimmunoassay for HLA antigens (Miyakawa *et al.*, 1972). However, the procedure cannot be considered to be a strictly quantitative assay of circulating TSTA in the serum because of the relatively high background and the interference by normal serum components (Fig. 8). But the data shown in Fig. 9 and 10 probably provide a general representation of the changes in the level of circulating TSTA in the serum of mice following inoculation of SL2 lymphoma cells. The pattern is remarkably similar to that found in the related investigation from this laboratory (Thomson *et al.*, 1973) in which levels of circulating TSTA in the serum of rats growing a syngeneic chemically-induced sarcoma were followed. Immediately after inoculation of $10^6$ SL2 cells TSTA appears in the blood, probably due to the autolysis of the majority of the tumour cells at the site of injection. The persistence of circulating TSTA in the blood both for the SL2 lymphoma and the sarcoma appears to be only a few days and the TSTA levels build up again as the tumour grows. Following surgical removal of the tumour, the circulating

TSTA disappears from the blood within a few days.

It is noteworthy that the procedures used for the radioimmunoassay in this study and in the rat sarcoma investigations were very different in that (1) the source and the procedure used for the isolation and purification of the TSTA were quite dissimilar; (2) the specific antiserum in the sarcoma experiments was derived from syngeneic animals as opposed to the absorbed alloantiserum employed in the present study; (3) the radioimmunoassay in the sarcoma studies involved binding to insolubilized antiserum as opposed to precipitation by a xenogeneic anti-mouse IgG in the SL2 experiments. That both studies showed a similar pattern for the changes in the blood levels of TSTA during tumour growth gives confidence that the observations are genuine.

The present assay is not sufficiently precise to determine whether the serum of tumour bearing mice contains antibodies that bind soluble TSTA. However, the finding that there is no significant increase in the level of TSTA in sera after they have been treated so as to dissociate possible antigen–antibody complexes indicates that at most a small fraction of the TSTA released by the growing tumour is complexed with antibody. Nonetheless, it would appear that the DBA2 mice are capable of producing antibodies directed against the TSTA of SL2 lymphoma since the serum from DBA2 mice which have been hyperimmunized with irradiated SL2 cells binds the $^{125}$I-TSTA isolated from the ascitic fluid. An interesting finding is that this hyperimmune serum does not lyse SL2 cells in the presence of complement and studies are in progress to determine the immunoglobulin classes of this antiserum.

This investigation has been supported by a Programme Grant of the Medical Research Council.

### REFERENCES

EGAN, M. L., LAUTENSCHLEGER, J. T., COLIGAN, J. E. & TODD, C. W. (1972) Radioimmune Assay of Carcino-embryonic Antigen. *Immunochemistry*, **9**, 289.

HUNTER, W. M. (1974) Preparation and Assessment of Radioactive Tracers. *Br. med. Bull.*, **30**, 18.

LOWRY, O. H., ROSEBROUGH, N. J., FARR, L. & RANDALL, R. J. (1951) Protein Measurement with the Folin Phenol Reagent. *J. biol. Chem.*, **193**, 265.

MIYAKAWA, Y., TANIGAKI, N., YAGI, Y. & PRESSMAN, D. (1972) Determination of Human Histocompatibility Antigens in the Peripheral Blood by Radioimmunoassay. *Transplantation*, **13**, 481.

SANDERSON, A. R. & WELSH, K. I. (1972) Purification and Structural Studies of Alloantigen Determinants Solubilized with Papain. In *Transplantation Antigens.* Ed. D. Kahan and R. A. Reisfeld. New York and London: Academic Press. p. 273.

THOMSON, D. M. P., SELLENS, V., ECCLES, A. & ALEXANDER, P. (1973) Radioimmunoassay of Tumour Specific Transplantation Antigen of a Chemically Induced Rat Sarcoma: Circulating Soluble Tumour Antigens in Tumour Bearers. *Br. J. Cancer*, **28**, 377.

WELSH, K. I. & SANDERSON, A. R. (1974) Properties of Histocompatibility (HL-A) Determinants. II. Soluble Antigen-antibody Complexes. *Transplantation*, **17**, 290.

WIGZELL, H. (1965) Quantitative Titrations of Mouse H-2 Antibodies using $^{51}$Cr-labelled Target Cells. *Transplantation*, **3**, 423.

WOLF, A., BARFOOT, R. K. & JOHNSON, R. A. (1972) Xenogeneic Recognition of Tumour Specific Plasma Membrane Antigens from Mouse Lymphoma Cells. *Immunology*, **22**, 485.

WOLF, A. & STEELE, K. A. (1975) Separation of a Tumour Specific Transplantation-type Antigen from the Ascitic Fluid of Mice bearing a Syngeneic Lymphoma. *Br. J. Cancer*, **31**, 684.